# Media for advocacy of mental health in the Ethiopian context. Current practice, gaps, and future directions

**DOI:** 10.3389/fpsyt.2023.1248827

**Published:** 2023-08-24

**Authors:** Mohammed N. Anbessie, Yonas L. Belete, Biniyam A. Ayele, Victor Valcour, Niall Kavanagh, Caroline Prioleau, Kalkidan Yohannis

**Affiliations:** ^1^Global Brain Health Institute, University of California, San Francisco, CA, United States; ^2^Amanuel Mental Specialized Hospital, Department of Psychiatry, Research and Training Directorate, Addis Ababa, Ethiopia; ^3^Department of Neurology, College of Health Science, Addis Ababa University, Addis Ababa, Ethiopia; ^4^Memory and Aging Center, Medical Center, University of California, San Francisco, CA, United States; ^5^SWEDESD – Department of Women’s and Children’s Health, Uppsala University, Uppsala, Sweden; ^6^WOMHER – Women’s Mental Health During the Reproductive Lifespan, Interdisciplinary Research Center, Uppsala University, Uppsala, Sweden; ^7^Department of Psychiatry, College of Health and Medical Science, Dilla University, Dilla, Ethiopia

**Keywords:** media, advocacy, mental health, Ethiopia, social media

## Abstract

Media plays a crucial role in reshaping societal attitudes and behaviors towards individuals with mental illness. It contributes to improved rights of people living with mental health conditions and access to care services. However, in Ethiopia, mental health advocacy faces obstacles such as deep-rooted misconceptions, fear, and discrimination about mental illness, as well limited engagement of stakeholders and language barriers. Both mainstream and social media play a large role in disseminating mental health topics in Ethiopia. However, they need organized initiatives and efforts in order to be successful in promoting mental health awareness to the public. Implementing a comprehensive strategy comprising public awareness campaigns, policy advocacy, community engagement, stakeholder collaboration, responsible reporting, and increased coverage of mental health topics is crucial. The World Health Organization also emphasizes the role of health ministries in supporting mental health advocacy efforts. By promoting education, challenging stigmas, and improving access to mental health services, media advocacy can contribute to creating a more informed and supportive society for individuals with mental illness in Ethiopia.

## 1. Background

### 1.1. Importance of mental health advocacy and its impact on individuals and communities

Mental health advocacy is crucial in promoting the well-being of individuals and the community. It usually starts when family members or caretakers start to raise their voices, followed by people living with mental illness and later health workers, non-governmental organizations (NGOs), advocates, and government bodies. Strong advocacy movements are considered a motor for change and can substantially influence policymakers and legislators (1). According to the World Health Organization (WHO), mental health advocacy plays a crucial role in reshaping the social determinants of mental health by promoting individual rights and empowerment in their communities as well as by bringing equity in health care access (2).

Advocacy helped bring a positive impact in changing societal attitudes and behaviors towards individuals with mental illness, which in turn improved their social integration and access to necessary support. For example, mental health advocacy movements in developed countries have not only transformed societal perceptions of individuals with mental disorders, but also enabled mental health service users to have a stronger voice in articulating their needs and making informed decisions about their treatment, leading to positive outcomes such as improved policies, changes in laws and regulations, enhanced mental health promotion and prevention, protection of rights, and advancements in mental health services and care (3). Additionally, people familiar with mental illness tend to be more empathetic and compassionate and more helpful toward people living with mental illness (4).

In African countries, such as Ethiopia, mental health advocacy faces obstacles due to deep-rooted misconceptions, fear, and discrimination about mental illness. Challenges are further compounded by cultural beliefs, practices, and language barriers, which are often reinforced by strong community connections. Moreover, the valuable role of traditional healers and religious leaders in enhancing the well-being of individuals with mental illness is frequently neglected in advocacy campaigns (5). Effective mental health advocacy and health promotion in Africa require patience, perseverance, determination, and the establishment of supportive alliances.

Media plays a pivotal role in shaping public perceptions and attitudes toward mental health, with the potential for both positive and negative impacts. The responsible reporting of mental health can bring about numerous benefits, including transforming public perspectives and preventing mental health issues such as suicide and substance use disorders (6). However, irresponsible media portrayals can reinforce stigma, stereotypes, and misinformation surrounding mental health conditions. By presenting mental health issues responsibly and compassionately, the media can contribute to reducing the stigma and cultivating a society that is more supportive and well-informed.

## 2. Current practice of media advocacy for mental health in Ethiopia

### 2.1. Mainstream media platforms in Ethiopia

In Ethiopia, mental health topics are disseminated through two primary categories of media platforms. The first category encompasses traditional media, including print media (such as newspapers, magazines, books, billboards, and fliers) and broadcast media (such as television, radio, and film). The second platform is referred to as “new media” or social media, which encompasses popular platforms like Facebook, YouTube, TikTok, Twitter, and Instagram.

In the realm of broadcast media, Ethiopia is home to federal and state government-owned broadcasting agencies including, the Ethiopian Broadcasting Corporation (EBC), Fana Broadcasting Corporation (FBC), and Walta Media and Communication Corporate S. In recent years, privately owned broadcast corporations have also emerged, gradually establishing themselves within the mainstream media landscape. These privately owned entities are gaining traction, presenting competition to government-owned agencies. Table 1 shows some of the media agencies operating in Ethiopia.

**Table 1:** List of media in Ethiopia Source: https://ethiopianmediacouncil.org/council-members/.

Television and Radio	Television
Ethiopian Broadcasting Corporation	African Renaissance Television (Arts TV)
South Radio and TV Agency	TV 9 Ethiopian
Dire Dawa Mass Media Agency	Nahu Television
Fana Broadcasting Corporate	Ye Ethiopian Lijoch TV
Ahadu Radio and TV	EBS Television
Amahara Mass Media Agency	Walta Media and Communication Corporate
Harari Mass Media Agency	
Somali Mass Media Agency	Radio
Addis Media Network	
ESAT	Sheger FM 102.1 Radio
	Awassa University Radio
Publication	Awash Radio
	Benshangul Gumuz Mass Media Agency
Ethiopian Press Agency	Gambella Mass Media Agency
Ethiopian News Agency	One Love
Reporter Newspaper	Oya ya Multimedia
Capital Newspaper	
Fortune Newspaper	Association
Addis Admas Newspaper	
Adisinya Construction Magazine	Ethiopian Journalist Association
Taza Magazine	Ethiopian Media Women Association
National Construction Magazine	Wabi Publishers Association
Addis Maleda Newspaper and ethio Business Magazine	Ethiopian Sport Journalist Association
League sport Newspaper	Ethiopian Public Radio Association
Addis Geze Magazine	Amhara Regional Media Association
Gion Magazine	Oromia Regional Media Association
World Sport Newspaper	Broadcaster’s Association
Merfe Magazine	Hentset Association
Kale Amba Magazine	Ethiopian Culture and Tourism Journalist Association
Senke Magazine	Ethiopian Mass Media Professional Association
Kumneger Magazine	

In Ethiopia, the mainstream media primarily emphasizes a select range of program genres, including news, entertainment, sports, and talk shows. The majority of these mainstream media outlets are concentrated in the capital city of Addis Ababa, while the number of television and radio stations continues to experience growth and expansion across the country.

### 2.2. Role of social media in advocating mental health

Social media has had a profound global impact, not only competing with mainstream media but also gaining significant popularity among the masses. It has become a platform for lifestyle gurus, self-development coaches, and influencers who wield considerable influence. Unfortunately, the field of mental health has lagged in this realm, with many social media users mislabeling individuals with mental illness in a negative manner and employing culturally insensitive language. This presents a new challenge, as the battle for accurate mental health information now extends to social media influencers disseminating scientifically unsubstantiated misinformation.

However, it is not too late to address this issue. One critical aspect that needs improvement is the inclusion of individuals with mental illnesses as a primary stakeholder in the design, implementation, and monitoring process of social media campaigns. Representation matters, and by involving those with lived experiences, we can ensure that the content shared on social media platforms is accurate, sensitive, and relevant. Establishing dedicated websites, developing a strong social media presence, and providing interactive services are effective measures to enhance mental health education and support through these online channels. This proactive approach will contribute to countering misinformation, promoting mental health literacy, and fostering a more informed and inclusive social media environment.

### 2.3. Initiatives and efforts undertaken by media agencies to promote mental health awareness

In recent years, Ethiopia has increasingly recognized the significance of integrating mental health issues into the media landscape. Notably, there has been a surge in the coverage of mental health topics across mainstream media platforms, including print media, radio, television, and film industries, to effectively engage with their target audiences. While the scale of these efforts may not be on par with that of developed nations, the initiatives undertaken thus far hold promising prospects for further advancements in mental health awareness and education. Here are some noteworthy examples of the initiatives and efforts undertaken by the media:

- **Responsible reporting**:
  - In pursuit of responsible reporting, significant efforts have provided training and capacity-building opportunities to journalists and media professionals in Ethiopia. These initiatives aim to equip them with the necessary knowledge and skills to effectively cover mental health topics and communicate them to the public in a responsible and accurate manner. Notably, the Ethiopian Psychiatry Association has allocated a specific budget to provide short-term training to journalists working in mainstream media. The training covers proper terminology for mental health disorders, guidelines on addressing the public, harm reduction reporting methods, censorship considerations, and advisory principles. Furthermore, journalists are trained on engaging experts to provide insights on specific mental health-related subjects. To incentivize participation, journalists are encouraged to undertake the training and are further motivated to produce mental health-related programs upon completion. These initiatives collectively contribute to enhancing the quality and responsible reporting of mental health matters in Ethiopia’s media landscape.
- **Increased coverage of mental health topics**:
  - There is increased coverage of mental health topics in Ethiopia in the recent years, with both government-owned and private media agencies allocating more attention to this crucial subject. Print publications, radio, and television platforms now dedicate space and airtime for discussions, interviews, and informative content on mental health issues. Prominent examples include the popular radio shows *Erq Ma’ed* and *Ye Aemro Tena* which are broadcast live weekly by one of the largest national broadcasting corporations. Television outlets are broadcasting mental health day events and mini documentaries about mental health to their audiences. TV shows focusing on psychology topics are also gaining momentum, competing with other program genres. Additionally, many newspapers and magazines have dedicated sections for mental health and psychology topics, recognizing their significance.
  - The film industry in Ethiopia is also playing a significant role in covering mental health-related movies, marking a significant leap forward. These movies often center around disorders such as dissociative disorders, psychosis, depression, addiction, child and adolescent abuse, and trauma. The acting in these movies closely reflects the real-world clinical and social symptoms, contributing to a more authentic portrayal. For instance, the popular movies *Heryet*^1^ and *Selanci*^2^ have gained recognition for their sensitive exploration of the mental health struggles faced by individuals living with these disorders.
  - The collective efforts of media agencies, through increased coverage and diverse platforms, are creating a more informed and open discourse on mental health in Ethiopia. Such initiatives have the potential to raise awareness, reduce stigma, and promote understanding and empathy within the wider society (7).
- **Collaboration and partnerships**:
  - There is growing collaboration between media agencies and various stakeholders in Ethiopia, including the Ministry of Health, mental health organizations, and NGOs. These have been established to drive mental health awareness campaigns, public service announcements, and other initiatives. Recognizing the importance of financial support, media platforms working on health topics often approach mental health clinics or NGOs in the field to secure sponsorship for their programs. In return, the media helps promote the vision and services of these clinics or organizations to their audiences.
  - An exemplary instance of such collaboration is the Federal Ministry of Health purchasing prime-time slots on one of the largest media broadcasting corporations to air two-minute mental health segments. This mutually beneficial approach allows the media to effectively communicate mental health information while creating a platform for the ministry to reach a broader audience with its mental health initiatives. This collaborative approach not only enhances the dissemination of mental health awareness but also fosters meaningful partnerships between media agencies and key stakeholders in the mental health sector.

## 3. Gaps and challenges in media advocacy for mental health in Ethiopia

There is a deep-rooted stigma and discrimination against mental illness. People living with mental illness, relatives/careers/ and even mental health providers face discrimination and fear of stigma. As such many people choose to live and suffer in hiding from their communities. In sub-Saharan Africa, including Ethiopia, there is a strong lineage of beliefs associated between mental illness and supernatural beings (8). The public often interprets certain mental illnesses like depression as laziness and weakness, and psychosis as supernatural or spiritual possessions. Because of this people with severe and chronic psychiatric conditions are often subjected to spiritual or traditional healers. It is not unusual to witness people with mental illness on holy water treatment, near churches.

The battle in mental health advocacy is between discriminating historically long-standing mass-held beliefs against humanistic approaches to mental illnesses and mental health treatment. Cultural and societal norms, as well as misconceptions surrounding mental illnesses, hinder the progress of advocacy efforts. These beliefs often attribute mental health problems to spiritual or supernatural causes, witchcraft, or personal weakness, leading to misconceptions and negative attitudes. Such cultural perceptions contribute to the isolation, marginalization, and discrimination faced by individuals with mental illnesses. Overcoming these deeply rooted beliefs requires sustained advocacy efforts to raise awareness, challenge stereotypes, and promote accurate information about mental health.

Moreover, the existing challenge is exacerbated by the limited availability and accessibility of mental health services in Ethiopia, along with insufficient budget allocations for mental health. The scarcity of mental health professionals in the country creates a significant barrier to effective advocacy efforts and the provision of comprehensive care and health education to individuals in need. Insufficient budgetary support for mental health exacerbates these systemic challenges, impeding the progress of advocacy initiatives and hindering the improvement of mental health services and resources in the country.

## 4. Addressing the gaps and challenges

Addressing these gaps and challenges requires a multifaceted approach that encompasses public awareness campaigns, policy advocacy, community engagement, and collaboration with stakeholders (3). Advocacy usually starts when family members or caretakers start to raise their voices, followed by people living with mental illness and later health workers, NGOs working on mental health, advocates, and government bodies. Strong advocacy movements are considered a motor for change and can substantially influence policymakers and legislators (9).

Countries such as Brazil, Italy, Uganda, Australia, Mexico, Spain, and Mongolia have demonstrated effective practices in mental health advocacy. For instance, Brazil has implemented a psychosocial rehabilitation program, aiming to uphold the rights of individuals with mental disabilities. In Africa, Uganda has taken a leading role in mental health advocacy, with the Ugandan Schizophrenia Fellowship in Kampala supported by the World Fellowship for Schizophrenia. This fellowship program, led by caregivers of individuals with schizophrenia, provides home visit counseling and community health education, resulting in a significant reduction in mental health stigma. In Mongolia, where mental health advocacy was initially limited, the Ministry of Health initiated efforts to combat stigma and discrimination by distributing educational materials and engaging with the media (10). These examples highlight successful initiatives that contribute to the advancement of mental health advocacy in their respective contexts.

**Figure 1:**
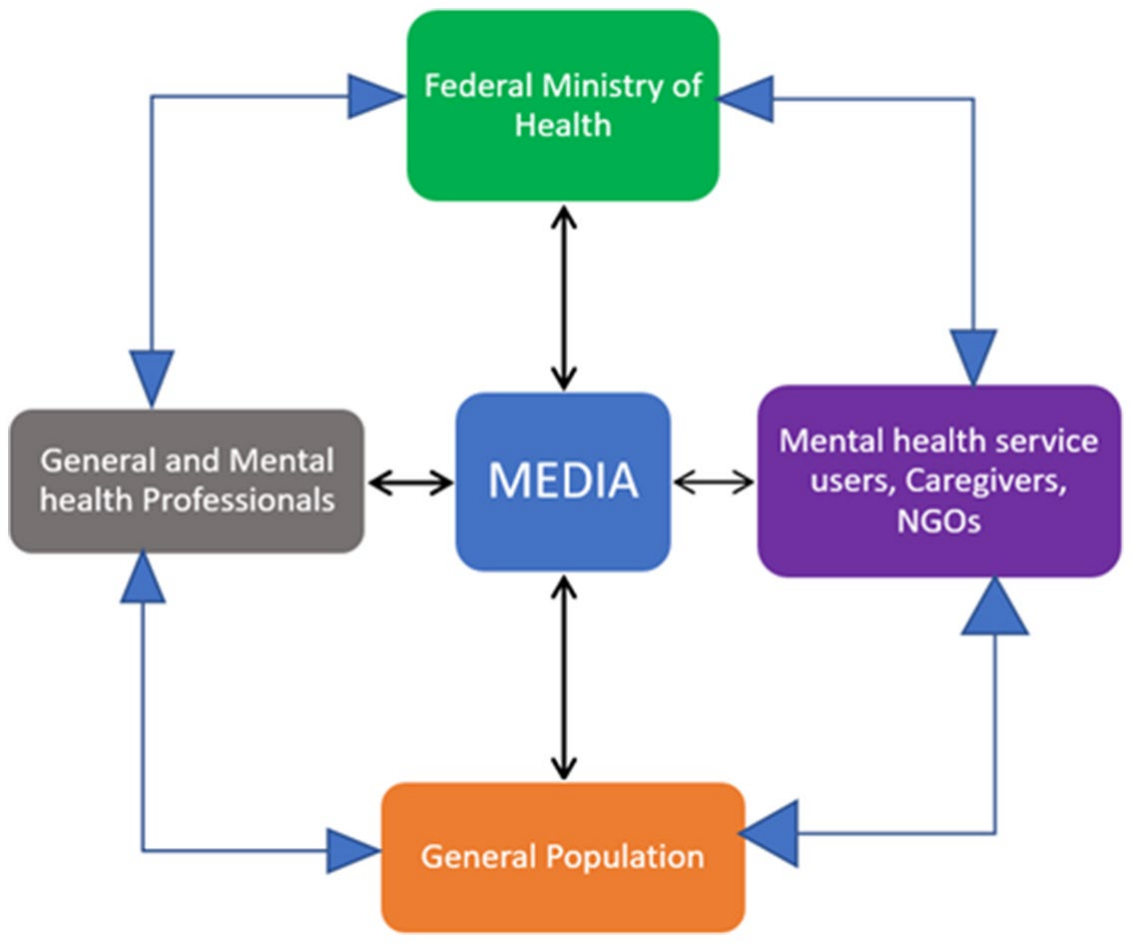
Stakeholder in mental health advocacy Adopted from WHO Advocacy for mental health.

The World Health Organization also urges the Ministry of Health of countries to support mental health advocacy works. They highlighted that health ministries should work jointly with policymakers, media, general and mental health professionals as well as the general population and mental health service users (10) (Figure 1). By promoting education, challenging stigmas, and advocating for improved services and policies, mental health advocacy in Ethiopia can strive towards fostering a more compassionate and inclusive society.

## 5. Conclusion

The objective of mental health advocacy is to strive for accessible and high-quality mental health services, recognizing that mere availability is not enough without increased service utilization. Advocates play a crucial role in promoting the integration of mental health policies within the broader healthcare system and advocating for the rights and well-being of individuals with mental illnesses. Cultural sensitivity and awareness are paramount in mental health education, necessitating the use of plain language and eliminating jargon while maintaining scientific accuracy. As Sir William Osler emphasized, the focus should be on taking mental health education to individuals, sharing lived experiences that inspire hope and exemplify the effectiveness of modern mental health treatments, thereby dispelling societal myths and misconceptions.

## Data availability statement

The original contributions presented in the study are included in the article/supplementary material, further inquiries can be directed to the corresponding author.

## Author contributions

MA contributed to the conception of the perspective piece and wrote the first draft of the manuscript. MA and YB contributed to the writing and organizing of the perspective piece. VV, NK, CP, and KY contributed to the review and revision of the perspective piece. All authors contributed to the article and approved the submitted version.

## Conflict of interest

The authors declare that the research was conducted in the absence of any commercial or financial relationships that could be construed as a potential conflict of interest.

## Publisher’s note

All claims expressed in this article are solely those of the authors and do not necessarily represent those of their affiliated organizations, or those of the publisher, the editors and the reviewers. Any product that may be evaluated in this article, or claim that may be made by its manufacturer, is not guaranteed or endorsed by the publisher.

## Footnotes

^1^ https://youtu.be/0MaZcR2_zZg

^2^ https://www.youtube.com/watch?v=x7EnIv5RgsE&t=950s
